# Evolution of Structure and Magnetism in FeCl_2_ and FeCl_3_: From Clusters to Monolayers

**DOI:** 10.1021/acs.jpca.5c05632

**Published:** 2025-12-01

**Authors:** Mehmet Emin Kilic, Manish Kumar Mohanta, Puru Jena

**Affiliations:** Physics Department, 6889 Virginia Commonwealth University, Richmond, Virgina 23284, United States

## Abstract

In this work, we address one of the most fundamental questions in cluster science—how do the structure and properties evolve from clusters to crystals? Using density functional theory (DFT), we focus our study on the evolution of structure and magnetism in iron-chloride systems, from clusters to monolayers. The choice of this system is motivated by the recent experimental confirmation of one of the author’s earlier theoretical prediction that the FeCl_2_ cluster is magnetic with a spin magnetic moment of 4 μ_B_ localized at the Fe site, while its dimer, Fe_2_Cl_4_, is antiferromagnetic. Similarly, FeCl_3_ cluster is magnetic with a total spin magnetic moment of 5 μ_B_, with 4 μ_B_ localized at the Fe site and 1 μ_B_ distributed over the Cl sites. The dimer clusters Fe_2_Cl_4_ and Fe_2_Cl_6_ have an antiferromagnetic ground state, and upon Li-functionalization, both can be magnetically transformed from antiferromagnetic to ferromagnetic states. In contrast, FeCl_2_ and FeCl_3_ monolayers exhibit different magnetic ground states in their periodic forms: FeCl_2_ is ferromagnetic (FM), but in FeCl_3_, the antiferromagnetic (AFM) and FM states are energetically nearly degenerate. Such a difference arises due to the different chemical coordination of the Fe atoms with the Cl atoms, caused by their different oxidation states, which is +2 in FeCl_2_ and +3 in FeCl_3_, respectively. Interestingly, Li-functionalization allows both FeCl_3_ and FeCl_2_ monolayers to be ferromagnetic. Our study highlights that several, but not all, electronic and magnetic characteristics of isolated clusters are preserved in the extended periodic structures. This systematic investigation of iron-halide clusters is expected to inspire further experimental and theoretical exploration into the magnetism of other transition metal halides.

## Graphical abstract

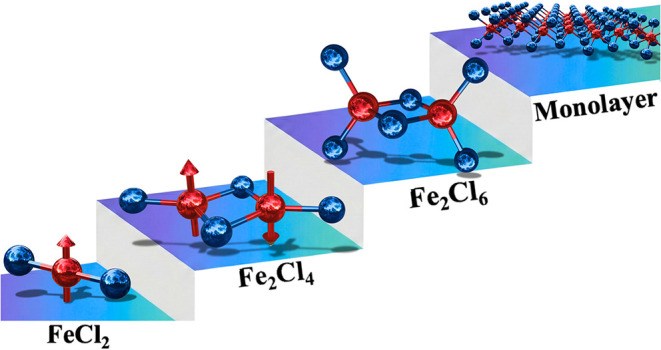

## 1 Introduction

Studies of atomic clusters as a function of size, composition, and structure have attracted considerable attention not only because their properties are unique, but also, they bridge our understanding of how properties evolve from clusters to crystals, one atom at a time.^1^ In this paper, we focus on the evolution of magnetism, from clusters to monolayers in FeCl_2_ and FeCl_3_ systems. Note that although half of the elements in the periodic table have a nonzero magnetic moment, there are only six elemental ferromagnets—Fe, Co, Ni, Gd, Dy, and Tb. In contrast, clusters of nonmagnetic elements with a suitable size and composition can be magnetic. One of the early studies that brought this into focus is that of a Li_4_ cluster whose ground state geometry is planar with singlet spin but has a magnetic moment of 2 μ_B_ when confined to a three-dimensional tetrahedral geometry.^2^ Later studies showed that V^3,4^ and Rh^5^ which are nonmagnetic in the bulk, can become magnetic in small clusters. In addition, the magnetic moments of clusters are also enhanced over their bulk values.^6^ These properties, predicted by theory, have been confirmed experimentally.^7,8^

A decade ago, Pradhan and Jena predicted^9^ that FeCl_2_ cluster is magnetic with a spin magnetic moment of 4 μ_B_ which is localized at the Fe site. Here, the two 4s-electrons of Fe (4d^6^ 4s^2^) bind with the Cl atoms, leaving its magnetic moment same as it is in the free atom. A dimer of FeCl_2_, i.e., Fe_2_Cl_4_, was shown to be antiferromagnetic with the Fe atoms retaining their atomic magnetic moment but aligned in opposite directions. This prediction was recently confirmed in an experiment by Kresin and co-workers^10^ who embedded the FeCl_2_ and Fe_2_Cl_4_ clusters in a liquid helium drop and measured their magnetic deflection in a Stern-Gerlach field. In contrast, crystalline FeCl_2_ is a weakly coupled van der Waals system where the intralayer coupling is ferromagnetic, but the interlayer coupling is antiferromagnetic^11^ with the magnetic moment at the Fe site remaining the same as it is in the free atom, namely 4 μ_B_. The FeCl_3_ cluster was also predicted^12^ to be magnetic with a magnetic moment of 5 μ_B_, with the iron atom carrying a magnetic moment of 4 μ_B_ and 1 μ_B_ distributed among the Cl atoms. Like Fe_2_Cl_4_, Fe_2_Cl_6_ cluster is antiferromagnetic with each Fe atom carrying a magnetic moment of 4 μ_B_ but aligned in opposite directions. When doped with a Li atom, both LiFe_2_Cl_4_ and LiFe_2_Cl_6_ clusters become ferromagnetic as the magnetic coupling switches from superexchange to double-exchange.^9^

Magnetic properties of FeCl_2_ and FeCl_3_ monolayers are different, while the former is ferromagnetic, we find the latter to exhibit nearly degenerate antiferromagnetic and ferromagnetic states. The immediate question that arises is why the magnetic couplings in the FeCl_2_ and FeCl_3_ monolayers are different while they remain the same in (FeCl_2_)_2_ and (FeCl_3_)_2_ dimers. Here, we address this question through calculations based on density functional theory. In addition, we also study the effect of Li decoration on the magnetic properties of FeCl_2_ and FeCl_3_ monolayers and compare our results with earlier theoretical predictions on their cluster analogues.^9^

These studies on Fe-chloride systems may have implications for the entire transition metal halide families, which have become a hotly pursued system since the first theoretical prediction of ferromagnetism in CrI_3_ monolayer^13^ and its subsequent experimental confirmation.^14^ This interest is fuled by the fact that atomically thin two-dimensional magnetic materials have potential applications in storage and optical devices, sensors, and spintronics. In the following, we present our theoretical methods followed by results on (i) the electronic and magnetic properties of FeCl_2_ (FeCl_3_) and Li-functionalized FeCl_2_ (FeCl_3_) clusters as well as pristine and Li-functionalized FeCl_2_ and FeCl_3_ monolayers. The results are are summarized in Section 4.

## 2 Theoretical Methods

All calculations are carried out using spin-polarized density functional theory (DFT) as implemented in the Vienna Ab initio Simulation Package (VASP).^15,16^ The exchange-correlation interactions are treated using the Perdew–Burke–Ernzerhof (PBE) functional^17^ within the generalized gradient approximation (GGA), combined with the projector augmented wave (PAW) method.^17^ To account for on-site Coulomb interactions in localized orbitals, the Hubbard U correction (DFT + *U*) is applied using the simplified Dudarev approach,^18^ with the effective *U* parameter, *U*_eff_ = *U* – J, varied systematically (from 0 to 4 eV) to assess its influence on the electronic structure. Dynamical stability is evaluated using density functional perturbation theory (DFPT) as implemented in the Phonopy package.^19^ For isolated gas-phase clusters, a sufficiently large vacuum spacing (20 Å) is introduced along all three Cartesian directions (*x*, *y*, and *z*) to prevent spurious periodic interactions. In contrast, for monolayer systems, vacuum separation is added only along the out-of-plane (*z*) direction. van der Waals interaction is considered using the DFT-D3 dispersion correction method of Grimme.^20^ All atomic structures are fully relaxed until the total energy and atomic forces converged to thresholds of 10^–5^ eV and 0.01 eV/Å, respectively. Bader charge analysis is employed for the ground state structures to reveal general trends in charge transfers.^21^

## 3 Results and Discussion

### 3.1 Atomic and Electronic Structure and Magnetic Properties of (FeCl_2_)_*n*_ (*n* = 1, 2) Clusters, FeCl_2_ Monolayer, and Li-Coated FeCl_2_ Monolayer

In the following, we first present results on atomic and electronic structure as well as the magnetic properties of (FeCl_2_)_*n*_ (*n* = 1, 2) clusters and their monolayer, without and with Li coating. Similar results are presented for the FeCl_3_ system in Section 3.2.

#### 3.1.1 (FeCl_2_)_*n*_ (*n* = 1, 2) Clusters

Figure 1 presents the optimized atomic structures of (FeCl_2_)_*n*_ (*n* = 1, 2) clusters, which are consistent with previous calculations.^9^ The spin-polarized DFT calculations for FeCl_2_ indicate a linear geometry (Figure 1a), with an Fe–Cl bond length of 2.115 Å and a total spin magnetic moment of 4 μ_B_, primarily localized on the Fe atom. For the Fe_2_Cl_4_ cluster, structural optimizations were performed considering both ferromagnetic (FM) and antiferromagnetic (AFM) spin configurations. In agreement with the previous study,^9^ the magnetic moments of Fe atoms in the ground-state Fe_2_Cl_4_ cluster are aligned in opposite directions (AFM coupling), which is 0.06 eV below the FM state with a total magnetic moment of 8 μ_B_. The coordination environment of chlorine varies significantly across the iron chloride clusters. In FeCl_2_, each chlorine atom is singly coordinated, forming discrete Fe–Cl bonds. However, in the Fe_2_Cl_4_ cluster, two distinct chlorine environments are identified: terminal chlorines bonded to a single Fe atom and bridging chlorines connected to both Fe centers. The coexistence of one- and two-coordinated chlorines imparts a mixed-valence character to the cluster and facilitates electronic communication between the Fe atoms, which in turn influences the overall electronic and magnetic properties. The Fe–Fe and Fe–Cl bonds in the ground state AFM configuration of Fe_2_Cl_4_ cluster are presented in Table 1. Note that while the AFM configuration is planar, the FM configuration adopts a nonplanar geometry (Figure 1b). This is similar to an earlier observation^22^ where the ground state of the Li_4_ cluster was found to be planar with zero spin, but in three-dimensional form, Li_4_ exhibits a spin magnetic moment of 2 μ_B_. These findings raise an important question: if Fe_2_Cl_4_ is to be considered as a functional molecular magnet, what is the energy barrier for the transitions between the FM to AFM states? As mentioned before, experimentally, Fe_2_Cl_4_ has been found to be antiferromagnetic,^10^ in agreement with the theoretical prediction.^9^

**Figure 1:**
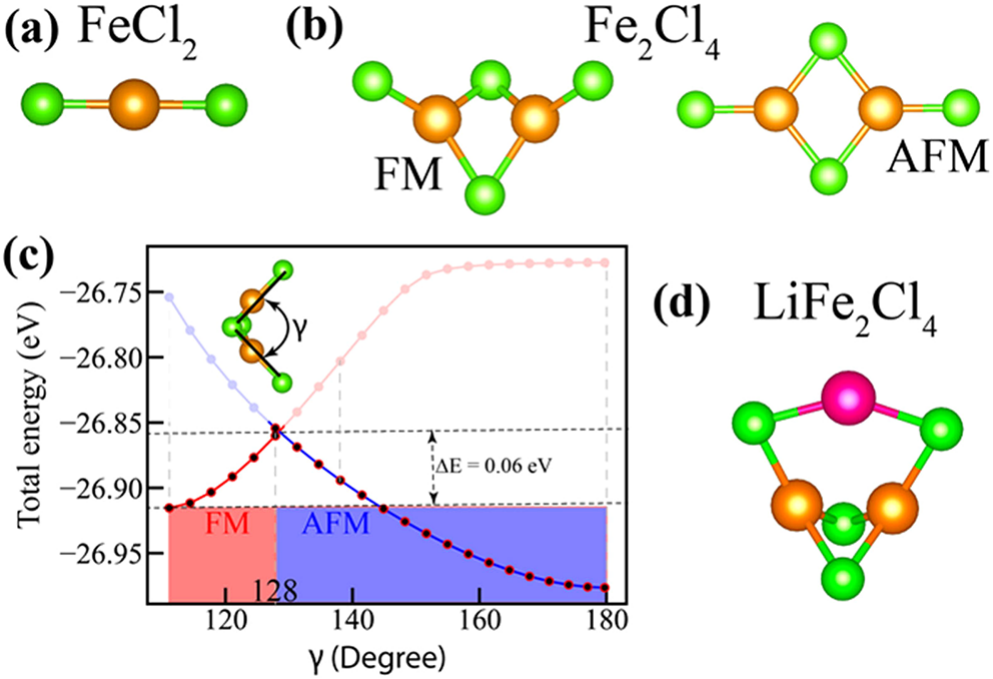
Optimized structure of the (a) FeCl_2_ and (b) Fe_2_Cl_4_ clusters in ferromagnetic (FM) and antiferromagnetic (AFM) configurations. (c) Total energy profile corresponding to the magnetic transition between FM and AFM states. (d) Optimized atomic structure of LiFe_2_Cl_4_ cluster. Orange, green, and pink spheres represent Fe, Cl, Li atoms, respectively.

**Table 1:** Optimized Fe–Fe and Fe–Cl Bond Lengths (in Å) for the Most Stable Spin Configurations of the Fe_2_Cl_4_, LiFe_2_Cl_4_, Fe_2_Cl_6_, and LiFe_2_Cl_6_ Clusters and Their Corresponding Monolayers^a^

		ground state	*d* (Fe–Fe)	*d* (Fe–Cl^a^)	*d* (Fe–Cl^b^)	Δ*E* ^PBE^	Δ*E* ^PBE+*U*^
Fe_2_Cl_4_	cluster	AFM	2.764	2.109	2.252	0.06	0.06
	monolayer	FM	3.536	-	2.470	–0.16	–0.02
LiFe_2_Cl_4_	cluster	FM	2.181	-	2.282	–1.10	–0.32
	monolayer	FM	2.563	-	2.239	–0.73	-
Fe_2_Cl_6_	cluster	AFM	3.051	2.132	2.302	0.19	0.06
	monolayer	FM/AFM	3.564	-	2.415	0.07	0.00
LiFe_2_Cl_6_	cluster	FM	2.878	2.136	2.278	–0.43	–0.02
	monolayer	FM	3.477	-	2.454	–0.31	–0.01

a The energy difference Δ*E* (in eV) between the lowest-energy FM and AFM states is also provided for both PBE and PBE + *U* functionals, where an effective *U* parameter (U* = *U – J*) of 4 eV is applied to Fe 3d orbitals. A positive Δ*E* value indicates that the AFM configuration is energetically favored. Cl^a^ and Cl^b^ denote one- and more-coordinated chlorine atoms bonded to other non-chlorine atoms (Fe, Li), respectively. Δ*E* = *E*(FM) – *E*(AFM), *E* is the total energy.

The energy barrier associated with the transition between two magnetic states (FM and AFM) is evaluated using spin-polarized DFT calculations. The FM state, corresponding to an out-of-plane configuration with a dihedral angle γ of 111°, was taken as the initial geometry, while the AFM state, associated with a planar configuration and a γ angle of 180°, served as the final geometry. To explore the transition pathway between these two states, we systematically varied the γ angle in 20° increments and computed the total energies for each intermediate structure in both FM and AFM states, while constraining both magnetic states to the same set of geometries. The results are presented in Figure 1c. As shown, the energy difference (*E*_FM_ – *E*_AFM_) remains negative up to a γ angle of 128°, indicating that the FM state is more stable in this range. Beyond this critical point, the energy difference becomes positive, favoring the AFM state. The energy required for transition from the FM state at γ = 111° to the AFM state at γ = 128° is calculated to be 0.06 eV, representing the energy barrier for the magnetic transition. This analysis also confirms that the AFM configuration is the true ground state beyond the critical γ angle.

The electronic structures, including the highest occupied molecular orbital (HOMO) and the lowest unoccupied molecular orbital (LUMO) molecular orbitals and energies are presented in Figure S1 of the Supporting Information (SI). Both spin-down and spin-up components exhibit distict orbital distributions, reflecting the magnetic asymmetry between the two Fe centers. The calculations reveal that LUMO and HOMO are depicted with energies of −4.79 and −5.63 eV, respectively. The spatial distributions of the HOMO are mainly localized on the Fe atoms, reflecting the dominant contribution of Fe-3d orbitals in the valence region. In contrast, the LUMO density is distributed along the Fe–Cl bonding framework, indicating notable Fe–Cl antibonding character and metal–ligand hybridization. These features suggest that Fe_2_Cl_4_ possesses a relatively narrow HOMO–LUMO gap, consistent with its partial delocalized electronic structure and potential magnetic activity.

#### 3.1.3 FeCl_2_ Monolayer

Thermodynamically, the most stable surface configuration of crystalline FeCl_2_ is along the (100)-Cl terminated^23^ direction (Figure 2a). The FeCl_2_ monolayer is composed of three atomic layers with the Cl atoms occupying the top and the bottom layers and the Fe atoms occupying the central layer. Since most transition metal compounds adopt either the *H*- or *T*- phase (Figure 2b,c), we evaluated the total energies of these two structural phases for FeCl_2_ and determined that the *T*-phase is energetically more favorable by 0.18 eV/unit cell compared to the *H*-phase. Based on this result, we focus our subsequent analysis on the *T*-phase FeCl_2_ monolayer. The optimized lattice constant of the FeCl_2_ monolayer in the *T*-phase is calculated to be 3.536 Å, which agrees well with previous result (3.5 Å).^11,23,24^ The phonon dispersion spectrum, as shown in Figure 2d, exhibits no imaginary frequencies across the Brillouin zone, confirming the dynamical stability of the structure. The spin-polarized electronic band structure of FeCl_2_ monolayer presented in Figure 2e reveals a significant spin asymmetry: spin-down states cross the Fermi level, indicating metallic behavior, whereas the spin-up channel exhibits a wide band gap of 4.40 eV, characteristic of semiconducting nature. This electronic asymmetry highlights the half-metallic character of the FeCl_2_ monolayer. The corresponding density of states (DOS) analysis in Figure 2e shows that the Fe *d*-orbitals predominantly contribute to the spin-down states near the Fermi level, further supporting the origin of the metallic behavior in one spin channel. To further examine the effect of electron correlation, we apply the Hubbard *U* correction to the GGA (PBE + *U*). Upon inclusion of the U* (*U – J*) term, the spin-up band gap increases to 4.61, 4.79, 4.95 eV for U* values of 1, 2, and 3 eV, respectively. This trend demonstrates the sensitivity of the spin-up band gap to on-site Coulomb interactions, while the metallic nature of the spin-down channel at the Fermi energy remains preserved (Figure 2e). We note that the FeCl_2_ monolayer exhibits 4 μ_B_/unitcell magnetic moment, localized at the single Fe atom in the primitive cell. We also evaluated the nonmagnetic configuration of FeCl_2_, which is found to be 0.91 eV/unitcell higher in energy than the ferromagnetic ground state. To further examine whether the antiferromagnetic coupling observed in Fe_2_Cl_4_ cluster persists in extended structures, we employed a 2 × 1 supercell containing two Fe and four Cl atoms. This setup allows for the arrangement of different spin configurations on the Fe atoms. Unlike the Fe_2_Cl_4_ cluster, the Fe_2_Cl_4_ monolayer is found to be ferromagnetic (both Fe atoms with parallel spins and each carrying 4 μ_B_), which is lower in energy than the AFM configuration (characterized by opposite spin orientations on the Fe atoms, +4 μ_B_ and −4 μ_B_), by 0.16 eV.

**Figure 2:**
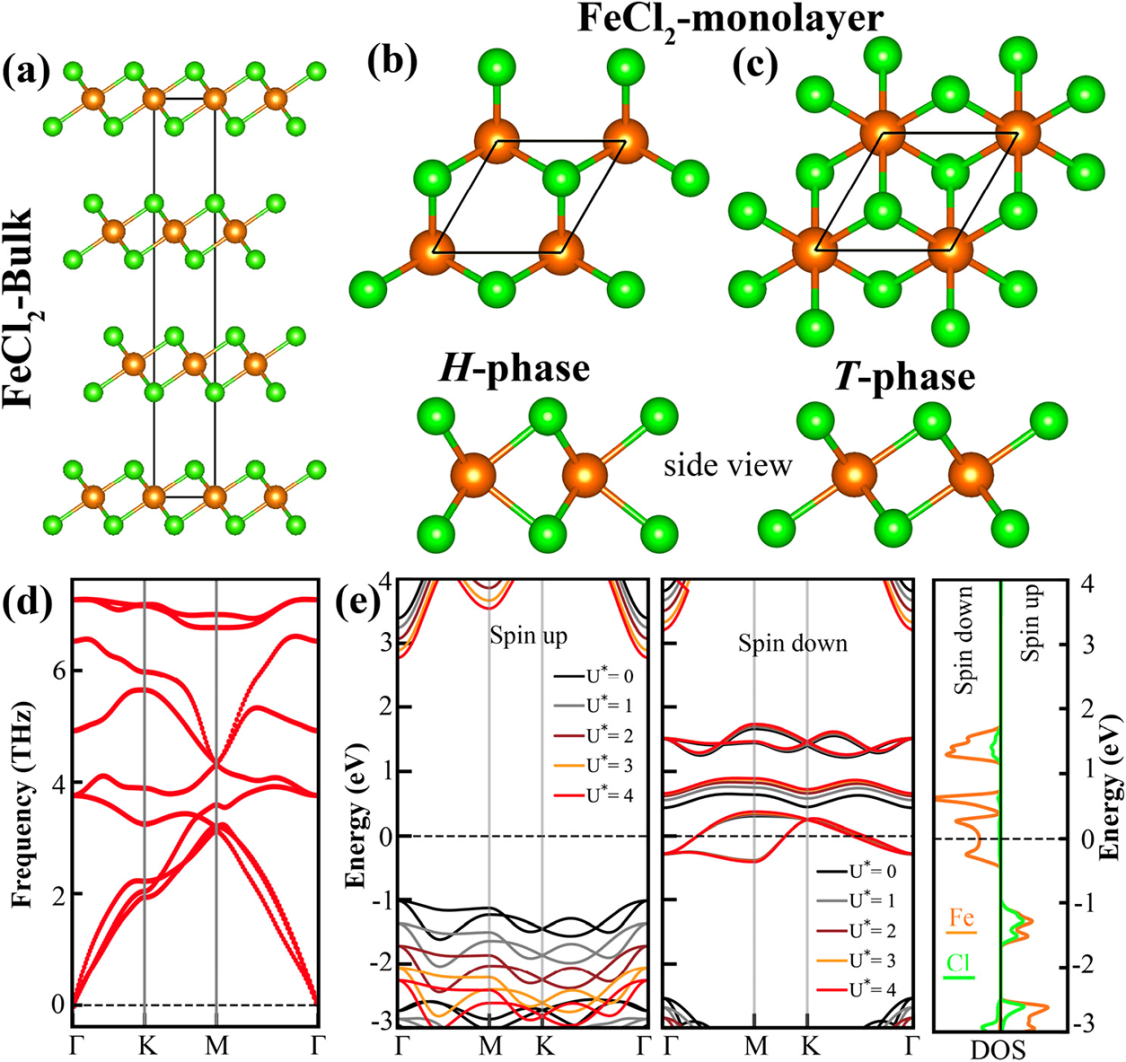
(a) Crystal structure of bulk FeCl_2_. Top and side views of the FeCl_2_ monolayer in (b) *H*- and (c) *T*-phase, with the unitcell outlined by a black rhombus. (d) Phonon dispersion spectrum of the FeCl_2_ monolayer. (e) Left panel: Spin-polarized electronic band structures of the FeCl_2_ monolayer calculated with different on-site Coulomb interaction values (U* = 0, 1, 2, 3, and 4 eV), shown in black, gray, brown, orange, and red, respectively. Right panel: Atom-projected density of states (DOS), with the Fermi energy set to zero. The DOS of Fe and Cl atoms are represented in orange and green, respectively.

While the Fe_2_Cl_4_ cluster favors an antiferromagnetic (AFM) ground state, the periodic monolayer stabilizes in a ferromagnetic (FM) configuration. This transition in magnetic ordering is influenced by changes in the coordination environment of Cl atoms. In the cluster, some Cl atoms are singly coordinated to Fe, whereas in the monolayer, lattice periodicity ensures that all Cl atoms exhibit similar coordination numbers, promoting magnetic symmetry and stabilizing the FM state. One way to understand this trend without constructing the full periodic monolayer is through atomic doping. Note that when a dopant atom, such as Li, was adsorbed onto the Fe_2_Cl_4_ cluster,^9^ it was found to bind symmetrically to two Cl atoms, effectively mimicking the bonding symmetry present in the periodic structure. Therefore, in the following, we discuss Li adsorption on the FeCl_2_ monolayer.

#### 3.1.4 LiFe_2_Cl_4_ Cluster and Li-Decorated FeCl_2_ Monolayer

Although LiFe_2_Cl_4_ cluster has previously been reported by Paradhan et al.,^9^ we revisited this system, as understanding Li adsorption is crucial for elucidating the changes in magnetic ordering that occur during the transition from cluster-based to periodic systems. Since Fe_2_Cl_4_ contains two distinct types of chlorine atoms—one coordinated to two Fe atoms and the other to a single Fe atom, we systematically explored all possible adsorption sites, including top, bridge, and hollow configurations, considering both in-plane and out-of-plane geometries (see Figure S2 of the SI). Calculations were performed for both FM and AFM spin states. The energetically most favorable configuration is shown in Figure 1d. In this structure, the Li atom preferentially binds to two chlorine atoms, each originally coordinated to only one Fe atom. Upon Li adsorption: (i) all chlorine atoms attain the same coordination number with elongated Fe–Cl bonds, which is critical for understanding the shift in magnetic preference between the cluster and periodic forms of Fe_2_Cl_4_; (ii) the geometry transitions from an in-plane to an off-plane configuration; and (iii) the ground-state magnetic ordering of Fe_2_Cl_4_ switches from AFM to FM. The energy difference between the FM and AFM configurations in the LiFe_2_Cl_4_ cluster is approximately 1.10 eV, indicating a robust FM ground state and demonstrating the potential of Li adsorption to induce magnetic switching in the Fe_2_Cl_4_ cluster.

The electronic structures of LiFe_2_Cl_4_ cluster, including the HOMO and LUMO molecular orbitals, their corresponding energies, and the Bader charge distributions, are presented in Figures S1 and S3 of the SI. Bader charge analysis reveals noticeable charge redistribution within the Fe_2_Cl_4_ cluster. Each Fe atom donates approximately 1.03 |e| to the surrounding Cl atoms, while the Cl sites act as electon-accepting centers, accumulating 0.49–0.53 |e| per atom. This electron transfer from Fe to Cl indicates significant Fe → Cl charge polarization, consistent with the higher electonegativity of Cl and the observed Fe–Cl bonding character. Likewise, in the LiFe_2_Cl_4_ cluster, Bader charge anlaysis indicates pronounced charge redistribution induced by the Li atom. The Li center donates approximately 0.88 |e| to the Fe–Cl network, consistent with its strong electropositive character. As a result, the Fe sites exhibit reduced electron density, carriying about 0.83–0.84 |e| each, while the Cl atoms act as electron acceptor, accumulating 0.58–0.70 |e| per site. This charge redistribution indicates that the electron introduced by Li is delocalized primarily over the Fe–Cl framework, resulting in a more evenly distributed charge density and slightly enhanced ionic character of the Fe–Cl interactions.

We further explored the magnetic properties of the Fe_2_Cl_4_ monolayer by decorating it with Li atoms. A single Li atom was placed at various adsorption sites within the Fe_2_Cl_4_ unitcell, as shown in Figure S4 of the SI. The total energy calculations were carried out for both FM and AFM spin configurations. The energetically most favorable configuration corresponds to the FM state, in which the presence of the Li atom breaks the perfect *T*-phase symmetry of the pristine FeCl_2_ monolayer. This lowest energy configuration is illustrated in Figure 3a. Consistent with the behavior of the LiFe_2_Cl_4_ cluster, total energy comparisons reveal that, in the Li-coated Fe_2_Cl_4_ monolayer, the FM configuration is approximately 0.73 eV more stable than the AFM configuration. Band gap analysis shows that the large gap in one spin channel slightly decreases to the range of 2.97–3.33 eV, while the gap in the other spin channel increases to 0.03–0.56 eV with the incorporation of Li, as shown in Figure 3b and Table S1 of the SI. The atom-projected density of states (DOS) indicates that Fe, Cl, and Li atoms contribute states near the Fermi energy.

**Figure 3:**
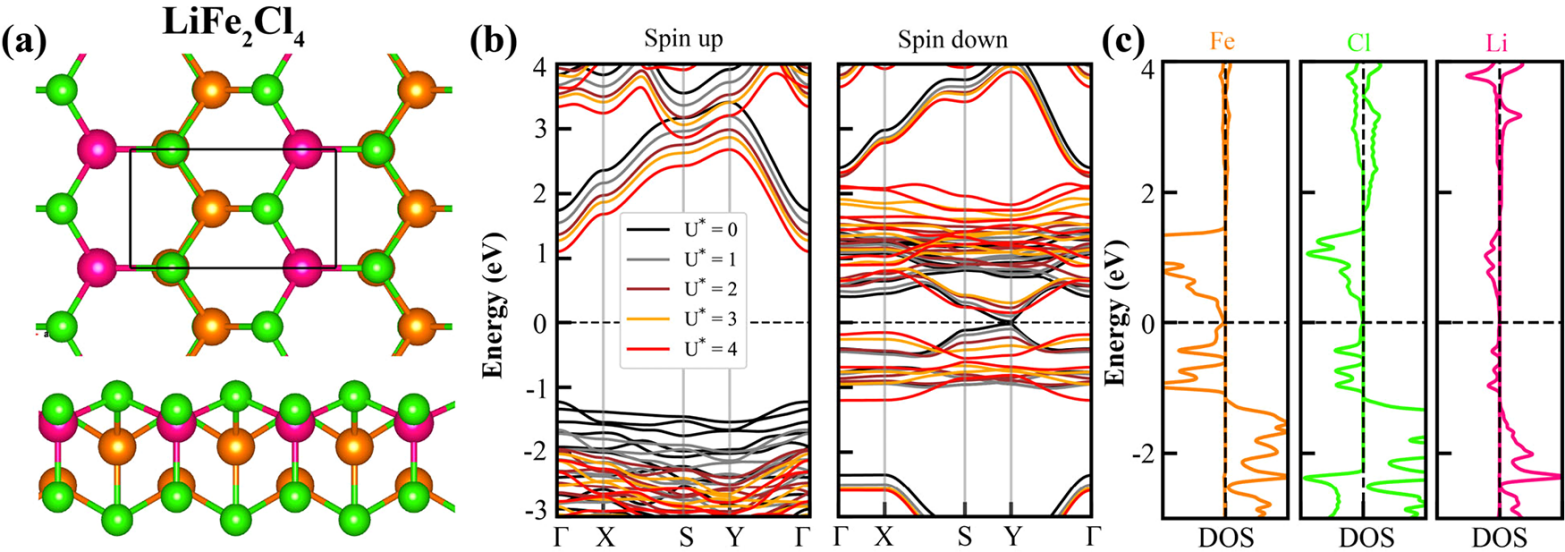
(a) Top and side views of the optimized atomic structure of LiFe_2_Cl_4_. (b) Spin-polarized electronic band structures for spin-up (left) and spin-down (right) channels, calculated using the PBE + *U* method with varying *U*_eff_ values. (c) Atom-projected density of states (DOS). Fermi energy set to 0 eV.

Returning to our main focus, we emphasize that the Li-coated Fe_2_Cl_4_ monolayer, similar to the LiFe_2_Cl_4_ cluster, stabilizes in a ferromagnetic ground state with lower total energy.

Although not the main focus of this work, we investigated the effect of Li adsorption on the symmetry of the FeCl_2_ *T*-phase monolayer. The addition of a single Li atom breaks the otherwise perfect symmetry of the layer. To explore whether this symmetry could be restored, we placed a second Li atom on the opposite side of the monolayer; however, structural distortions persisted despite this modification (Figure S5 of the SI).

### 3.2 Atomic and Electronic Structure, and Magnetic Properties of (FeCl_3_)_*n*_ (*n* = 1, 2) Clusters, FeCl_3_ Monolayer, and Li-Coated FeCl_3_ Monolayer

In the following, we present results on atomic and electronic structure as well as the magnetic properties of (FeCl_3_)_*n*_ (*n* = 1, 2) clusters and their monolayer, without and with Li coating.

#### 3.2.1 (FeCl_3_)_*n*_ (*n* = 1, 2) Clusters

The optimized geometries of the isolated FeCl_3_ and (FeCl_3_)_2_ clusters are shown in Figure S6 of the SI and Figure 4a, respectively. In the FeCl_3_ cluster, the Fe atom exhibits an oxidation state of +3. Of the total magnetic moment of 5 μ_*B*_ of the FeCl_3_ cluster, 4 μ_B_ is localized at the Fe-site, which is shown in the spin density plot in Figure S6 of the SI. The Fe_2_Cl_6_ cluster assumes a three-dimensional structure with two distinct types of Cl atoms, one type bridges two Fe atoms while the other is terminal, bonded to only a single Fe atom. The magnetic ground state of the Fe_2_Cl_6_ cluster is AFM, which is lower in energy than the FM state by 0.19 eV at the PBE and by 0.06 eV at the PBE + *U* level. A magnetic moment of ±4 μ_B_ is localized at the Fe-sites as illustrated in Figure 4b and the terminal (nonbridging) Cl-atoms exhibit a smaller magnetic moment of ± 0.23 μ_B_.

**Figure 4:**
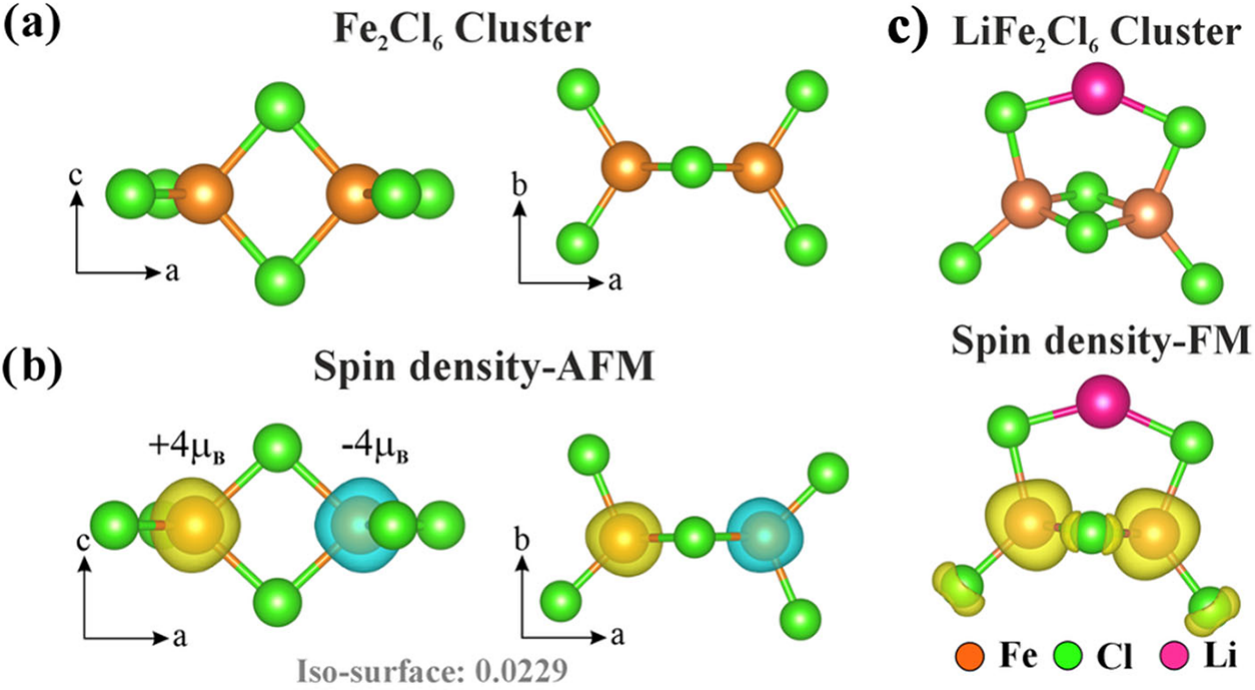
(a) Geometrical view of Fe_2_Cl_6_ cluster, (b) its spin density plot in AFM configuration, and (c) optimized geometry and spin density plot of isolated LiFe_2_Cl_6_ cluster.

#### 3.2.2 Electronic and Magnetic Properties of LiFe_2_Cl_6_ Cluster

The optimized geometry of the resulting LiFe_2_Cl_6_ cluster is shown in Figure 4c where the Li-atom binds to the previously terminal (nonbridging) Cl atoms. The spin-polarized calculation reveals that the LiFe_2_Cl_6_ cluster prefers a FM ground state, which is 0.43 eV lower in energy than the AFM configuration. This result demonstrates that Li adsorption is an effective strategy for tuning the magnetic state of the Fe_2_Cl_6_ cluster.

The electronic structures of LiFe_2_Cl_4_ cluster, including the HOMO and LUMO energies and Bader charge distributions, are presented in Figures S1 and S3 of the SI. The Fe_2_Cl_6_ cluster exhibits a HOMO–LUMO gap of 1.31 eV, while LiFe_2_Cl_6_ shows a reduced gap of 0.94 eV, implying that Li incorporation enhances electronic conductivity and reactivity. Bader charge analysis indicates that the Fe atoms in Fe_2_Cl_6_ donate approximately 1.30 |e| to the surrounding Cl atoms. Upon Li addition, the Fe centers become slightly less electron-donating, donating about 1.22 |e|, while the Cl atoms, particularly those bonded to Li, act as stronger electron acceptors, accumulating 0.65 and +0.66 |e|. The Li atom itself donates approximately 0.89 |e| to the cluster, confirming effective charge transfer. Overall, these results indicate that Li acts as an electron donor, particularly reducing the Fe centers and promoting enhanced delocalization of electron density across the Fe–Cl framework, consistent with the observed narrowing of the HOMO–LUMO gap.

#### 3.2.3 FeCl_3_ Monolayer

To enable a systematic comparison of the electronic and magnetic properties and to assess whether the behavior observed in isolated clusters persists in an extended periodic system, we investigated a monolayer of FeCl_3_. In this structure, the unit cell consists of two Fe-atoms and six Cl atoms, where each Fe^3+^ ion is octahedrally coordinated to six equivalent Cl atoms, forming edge-sharing FeCl_6_ octahedra. The top and side views of the optimized FeCl_3_ monolayer geometry are shown in Figure 5a. The phonon dispersion shown in Figure 5b confirms the dynamical stability of the FeCl_3_ monolayer. The magnetic ground state of FeCl_3_, at the PBE level of theory, is found to be antiferromagnetic. The energy difference between AFM and FM states is given in Tables 1 and S2 of the SI. It should be pointed out that several studies have been conducted on the FeCl_3_ monolayer, and while some have reported a ferromagnetic (FM) ground state,^25–27^ others have identified an antiferromagnetic (AFM) ground state.^28^ To understand this discrepancy, it should be noted that the energy differences between AFM and FM states are small. Thus, it is important to examine these differences by using different on-site Coulomb correlation (Hubbard *U*) as well as including van der Waal’s correction and determine if the true ground state of FeCl_3_ monolayer can be predicted within the accuracy of the calculatiions. We calculated the total energies with and without van der Waals corrections and for different values of the Hubbard *U* parameter (*U*_eff_ = 0, 3.6, 4 eV). These results are presented in Table S2 of the SI. For example, our results show that within the PBE + *U* framework, the energy difference between the FM and AFM configurations is nearly zero (*E*_FM_ – *E*_AFM_ = −0.0001 for *U*_eff_ = 4 eV without vdW). Thus, considering the intrinsic accuracy limits of DFT, it would be more appropriate to regard these two magnetic states as nearly degenerate.

**Figure 5:**
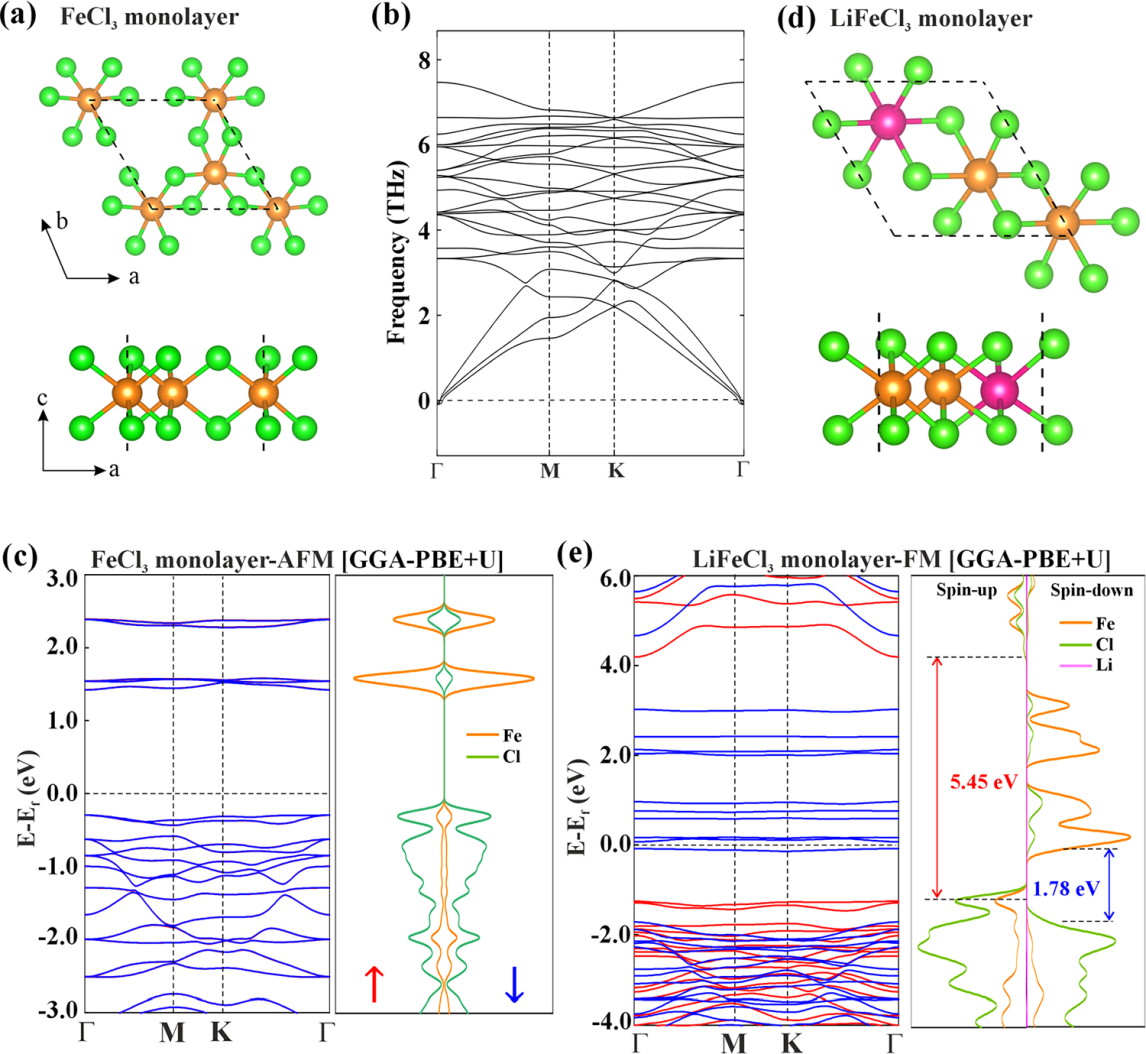
(a) Top and side view of FeCl_3_ monolayer, (b) phonon frequency plot, (c) electronic band structure, and atom projected density of states, (d) optimized geometry, and (e) electronic band structure analysis of LiFeCl_3_ monolayer.

For an isolated Fe-atom, the spin-only magnetic moment of Fe^3+^ (d^5^ configuration) is about $\mu_{s}=\sqrt{n\left(n+2\right)}$ μ_B_ ∼ 6 μ_B_; where n is number of unpaired electrons. However, in real solids such as FeCl_3_, a smaller local magnetic moment of ±4 μ_B_ is found at each Fe-site. Typically, the magnetic moments on Fe atoms tend to decrease as one goes from cluster to bulk.^29^ The magnetic moment of Fe atom varies in local environment, such as in previous report suggests that magnetic moment of Fe reduced from 2.72 μ_B_ per atom for pure Co matrix to 2.47 μ_B_ per atom for 75% Fe matrix.^30^ The magnetic moment of two Fe-sublatticee present in Fe_3_GeTe_2_ monolayers are reported to be 2.49 μ_B_ and 1.5 μ_B_.^31^ The electronic band structure indicates that the FeCl_3_ monolayer is a semiconductor with an indirect band gap. Recall that the FeCl_2_ monolayer is metallic. The atom projected band structure in Figure 5c reveals that the valence bands are primarily derived from Cl: p orbitals, while the conduction bands originate mainly from Fe: d orbitals, consistent with the electronic characteristics observed in the Fe_2_Cl_6_ cluster.

#### 3.2.4 Li-Decorated FeCl_3_ Monolayer

In the previous section, it was demonstrated that the antiferromagnetic Fe_2_Cl_6_ cluster can be turned into a ferromagnet via Li functionalization, mediated by the double-exchange mechanism. To see if the same manifests in a periodic crystal, a Li atom was adsorbed within the unit cell. There are two possible sites for Li-adsorption, which are shown in Figure S7 of the SI. The optimized unit cell of the energetically most favorable Li-adsorbed FeCl_3_ monolayer is shown in Figure 5d, where the Li-atom prefers the Fe-plane and is octahedrally coordinated to six equivalent Cl atoms. The optimized structure of Li-functionalized FeCl_3_ monolayer resembles that of the *T*-phase of FeCl_2_ monolayer, which has a ferromagnetic ground state. The spin-polarized DFT calculations confirm a magnetic transition from nearly degenerate AFM/FM to FM upon Li adsorption, with the FM configuration lying 0.31 eV lower in energy than that of the AFM state at the PBE level of theory. The electronic band structure of Li-doped FeCl_3_ monolayer is shown in Figure 5e, where the energy states near the Fermi level are predominantly contributed by Fe d orbitals. The spin-resolved band gaps are markedly different: the spin-up channel exhibits a wide band gap of 5.45 eV, while the spin-down channel shows a narrow gap of 1.78 eV, closely resembling the behavior observed in the Li decorated Fe_2_Cl_6_ cluster. A crystal structure with the composition Li–Fe–Cl, such as LiFeCl_4_, is very different from the Li-functionalized monolayer studied here.^32^ This work aims to demonstrate the magnetic transition of FeCl_3_ through Li functionalization.

Bader charge analysis shows that an electronic charge of 0.56 |e| is transferred from Li to the FeCl_3_ monolayer. A systematic comparison between the FM state of FeCl_3_ and LiFeCl_3_ in Figure 6 reveals that the spin-down conduction bands in LiFeCl_3_ monolayer are significantly lowered and cross the Fermi level, indicating a transition to metallic behavior. The FeCl_3_ monolayer remains geometrically robust under Li adsorption, although its electronic and magnetic properties undergo significant changes. Also, comparison of results in Sections 3.2.3 and 3.2.4 demonstrates that both the undoped and Li-doped FeCl_3_ monolayers retain the characteristic electronic and magnetic features of the corresponding molecular clusters.

**Figure 6:**
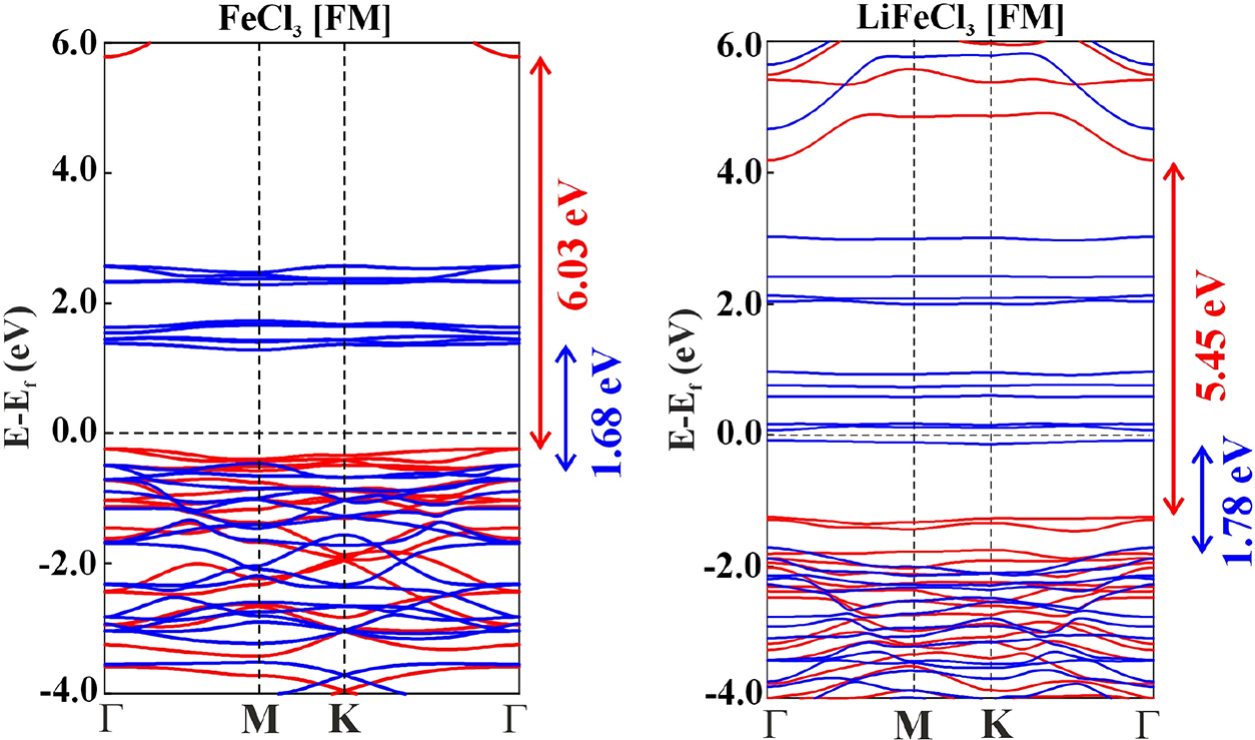
Comparative electronic band structure plot between FeCl_3_ (FM) and LiFeCl_3_ (FM).

## 4 Conclusion

Using spin-polarized DFT calculations, we have systematically investigated the electronic and magnetic properties of the iron-chloride systems (FeCl_2_, Fe_2_Cl_4_, and LiFe_2_Cl_4_ clusters; FeCl_2_ and LiFeCl_2_ monolayers; FeCl_3_, Fe_2_Cl_6_, and LiFe_2_Cl_6_ clusters; FeCl_3_ and LiFeCl_3_ monolayers) to examine how these properties evolve from clusters to monolayers. Except for the FeCl_2_ system, where its dimer, *i.e*., Fe_2_Cl_4_ cluster, is antiferromagnetic but the FeCl_2_ monolayer is ferromagnetic, the magnetic properties of the other iron-chloride systems studied here remain the same between the clusters and the monolayers. The underlying reason for the different magnetic behavior of the Fe_2_Cl_4_ cluster versus the FeCl_2_ monolayer is rooted in their structural difference. Note that the ground state structure of the Fe_2_Cl_4_ cluster is planar, while that of the FeCl_2_ monolayer is three-dimensional. As we have shown before, the planar antiferromagnetic Fe_2_Cl_4_ cluster turns ferromagnetic when the structure changes to a three-dimensional form. Note that in all other cases studied, the dimensionality of the clusters and monolayers remains the same. This observation is similar to that found in Li_4_ clusters 40 years ago.^22^ Here, the ground state of the Li_4_ cluster is planar and nonmagnetic, but it assumes a magnetic configuration with a magnetic moment of 2 μ_B_ when confined to a three-dimensional (tetrahedral) form. This dimensionality-driven magnetic transition can be exploited in synthesizing new magnetic memory devices.

While these studies are focused on the iron-chloride systems, they may have implications for the transition-metal halide systems in general. While many studies have been carried out on transition metal-halide monolayers and bulk structures^12,33–36^ similar studies are not available in clusters. For example, can the magnetic coupling between transition metal (X) atoms in X_2_Y_4_ and X_2_Y_6_ (X = 3d elements; Y = F, Br, I) be different from what is observed in the iron-chloride systems, depending upon the halogen ligands? In other words, could these X_2_Y_4_ and X_2_Y_6_ clusters be ferromagnetic with a suitable choice of X or Y atoms? If so, it may not be necessary to induce ferromagnetic order by doping metal atoms such as Li, as was found to be the case in iron chloride systems. This study provides a foundation for experimental and theoretical investigations into the magnetic properties of transition metal-halide systems, spanning from molecular clusters to extended periodic crystals. We should also point out that the Li atom was used as a dopant to induce a magnetic transition in both clusters as well as monolayers. We expect similar results if other alkali metal atoms are used as dopnats instead of Li.

## Supplementary Material



## Notes

Frontier molecular orbitals of the investigated clusters; possible adsorption sites for a Li atom on the Fe_2_Cl_4_ structure; Bader charge analysis; potential adsorption sites for a Li atom on the Fe_2_Cl_4_ monolayer; energetically favorable configuration of Li_2_Fe_2_Cl_4_; spin density distribution plot for the FeCl_3_ cluster; Li-adsorbed FeCl_3_ monolayer structure; electronic band gaps of the FeCl_2_ and LiFe_2_Cl_4_ monolayers; total energies of the FeCl_3_ monolayer (PDF)
