# Supplementary material for: Evolution of Structure and Magnetism in FeCl2 and FeCl3: From Clusters to Monolayers
Source: J Phys Chem A. 2025 Dec 1;130(6):1233–41. doi: 10.1021/acs.jpca.5c05632 (PMC12908144; doi:10.1021/acs.jpca.5c05632)
Supplement: Supplementary file 1 [file jp5c05632_si_001.pdf]

Supporting Information

Evolution of Structure and Magnetism in $FeCl_2$ and $FeCl_3$: From Clusters to Monolayers

Mehmet Emin Kilic[1], Manish Mohanta[2], and Puru Jena[*]

Physics Department, Virginia Commonwealth University, Richmond, Virginia, 23284

[1] and [2]: Equal contribution

[*]pjena@vcu.edu

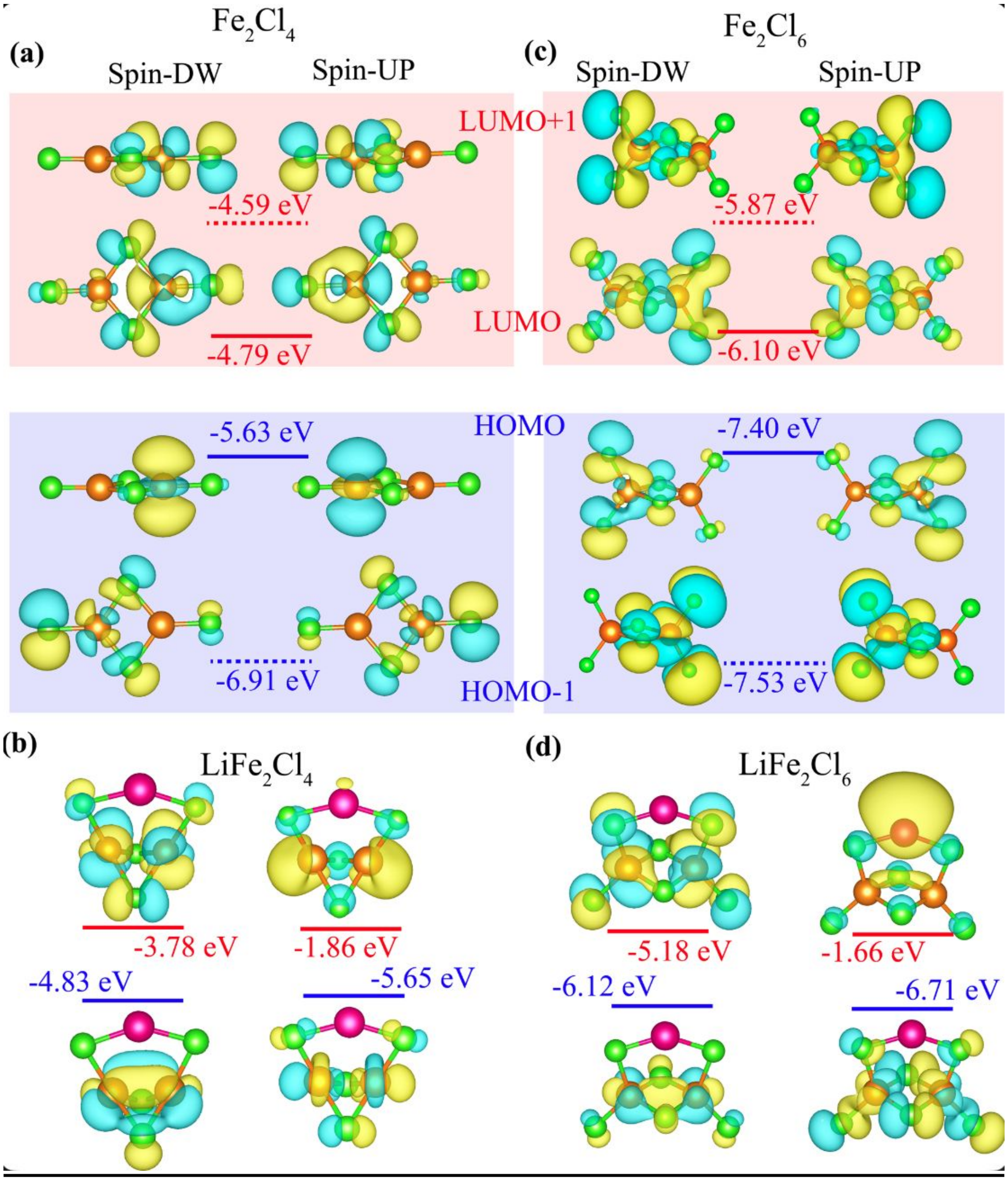

Figure S1 Frontier molecular orbitals of the ground state clusters (a) Fe_2Cl_4, (b) $LiFe_2Cl_4$, (c) Fe_2Cl_6, and (d) $LiFe_2Cl_6$. The figure displays the Highest Occupied Molecular Orbital (HOMO) and Lowest Unoccupied Molecular Orbital (LUMO) wavefunctions along with their corresponding energies.

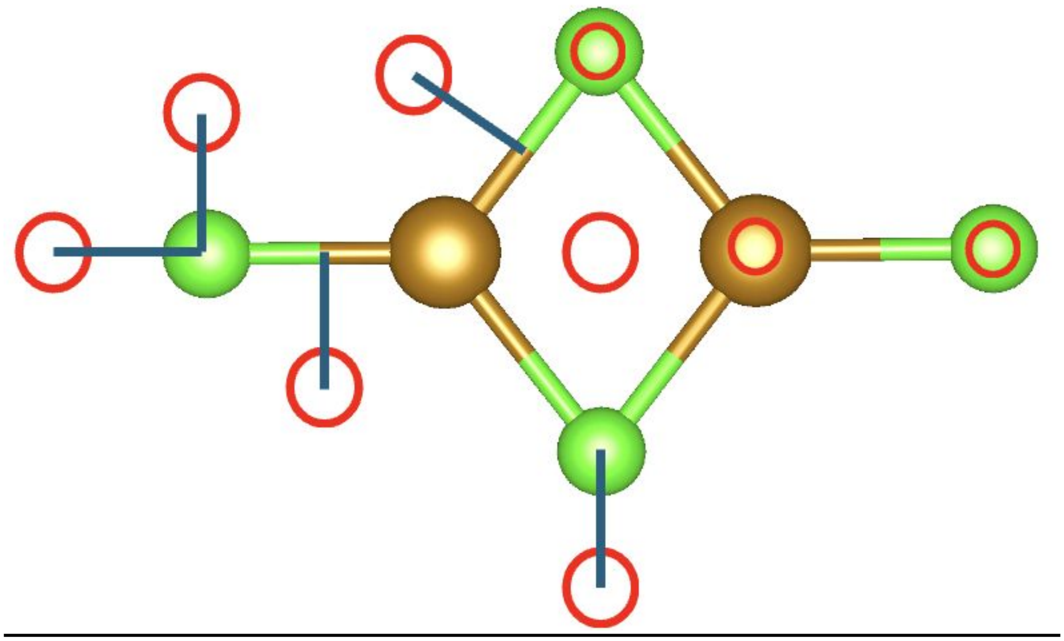

Figure S2 Possible adsorption sites for Li atom on the Fe_2Cl_4 cluster in the antiferromagnetic (AFM) configuration. The optimized in-plane Fe_2Cl_4 (AFM) structure was used as the initial configuration. Nine non-equivalent adsorption sites were identified, as indicated by the red circles. A single lithium atom was placed on these sites and structurally optimized in both FM and AFM states without any constraints. In total, 18 initial structures atom were evaluated, and the configurations with the lowest total energies for both FM and AFM states were selected.

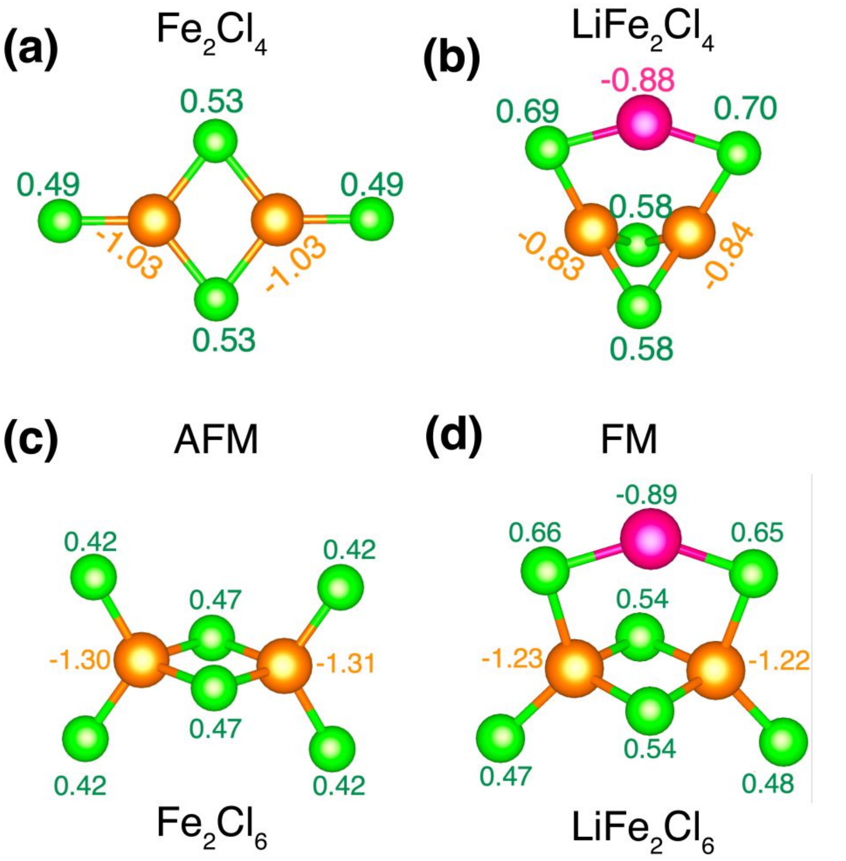

Figure S3 Bader charge analysis of (a) Fe_2Cl_4, (b) $LiFe_2Cl_4$, (c) Fe_2Cl_6, and (d) $LiFe_2Cl_6$. Orange, green, and pink spheres represent Fe, Cl, and Li atoms, respectively. The Bader charge values for each atom are indicated directly on the corresponding spheres, illustrating the charge distribution within the clusters. Positive and negative values indicate whether an atom donates or accepts electron density, respectively, highlighting the direction of charge transfer in each cluster.

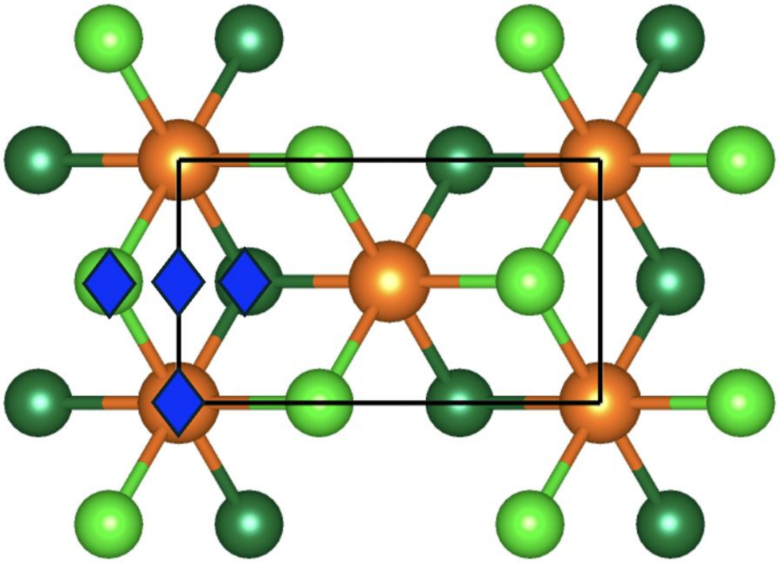

Figure S4 Possible adsorption sites for a single Li atom on the Fe_2Cl_4 monolayer. Four non-equivalent adsorption sites were identified: the top of the upper Cl atom (light green), the bottom Cl atom (dark green), the top of the Fe atom (orange), and the bridge site between two Fe or two Cl atoms, as indicated by the blue rhombus. A single Li atom was placed at each site and the structures were optimized in both ferromagnetic (FM) and antiferromagnetic (AFM) configurations without any constraints.

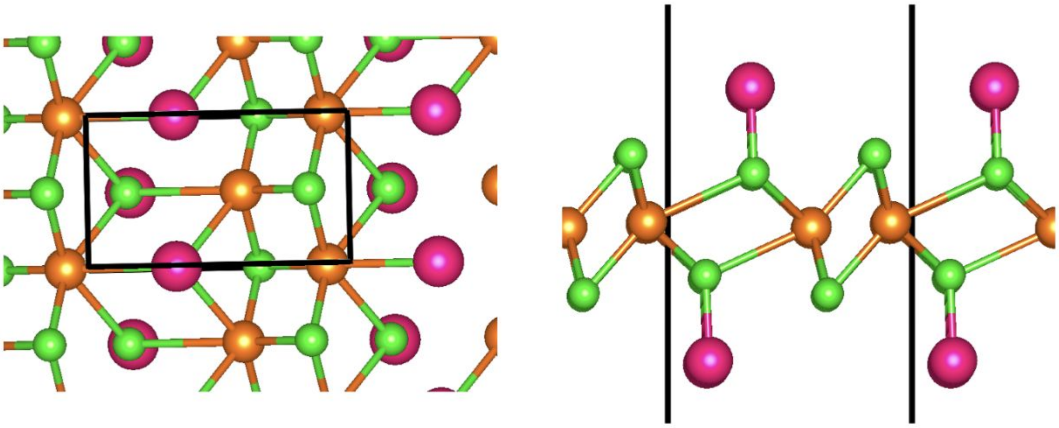

Figure S5 Top and side views of the energetically favorable configuration of $Li_2Fe_2Cl_4$ where Li, Fe, and Cl atoms are represented by pink, orange and green spheres, respectively.

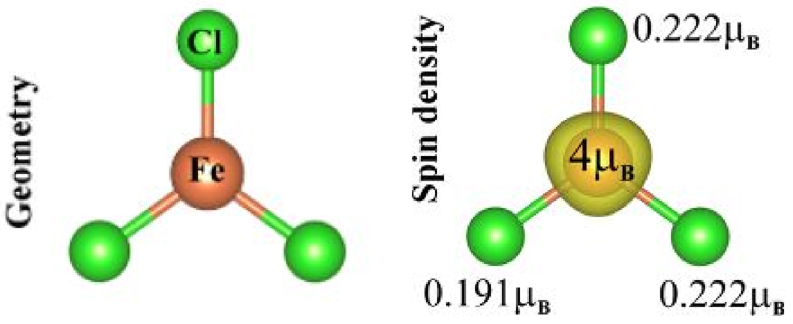

Figure S6 Geometrical view of $FeCl_3$ cluster and its spin density plot.

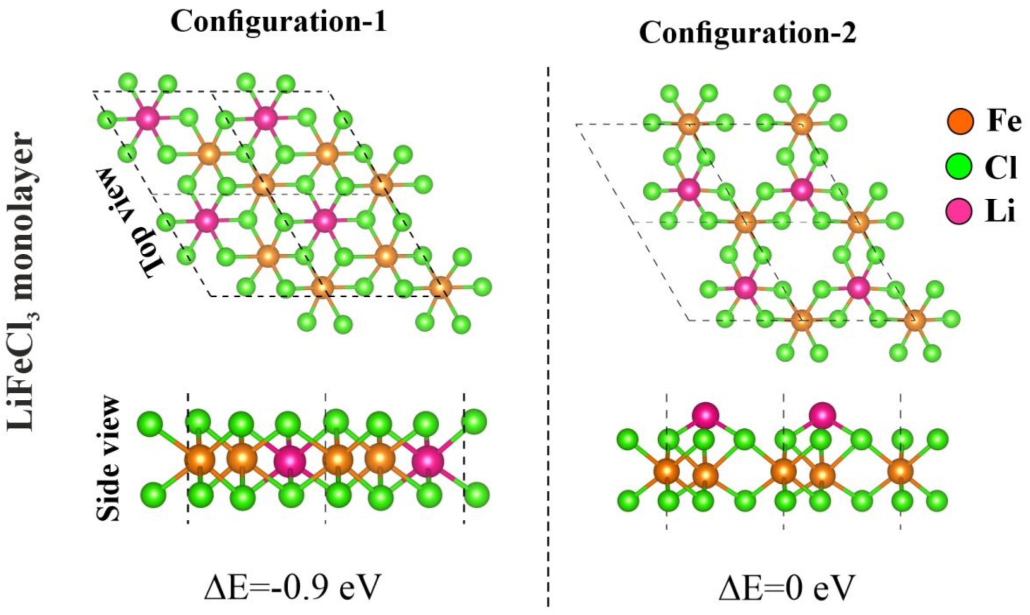

Figure S7 Different configurations of Li-adsorbed $FeCl_3$ monolayer system.

Table S1 Electronic band gaps of $FeCl_2$ and $LiFe_2Cl_4$ monolayers calculated using the PBE+*U* method, with U_{eff} values of 0, 1, 2, 3, and 4 eV. The band gaps for spin-up and spin-down channels are given separately.

	$FeCl_2$		$LiFe_2Cl_4$	
PBE + U	Egap		Egap	
U	Spin-UP	Spin-DOWN	Spin-UP	Spin-DOWN
0	4.36	-	2.97	0.03
1	4.45	-	3.20	0.20
2	4.53	-	3.33	0.46
3	4.69	-	3.30	0.56
4	4.84	-	3.23	0.31

Table S2: Total energies in eV of the $FeCl_3$ monolayer in FM and AFM magnetic states, calculated with and without van der Walls corrections, and for different U_{eff} values (0, 3.6, and 4.0 eV). The van der Walls interactions were included using the DFT-D2 method (*J. Comput. Chem*. 27, 1787 (2006) and the DFT-D3 method (*J. Chem. Phys.* 132, 154104 (2010))

	**PBE+*U* (U_{eff}=0)**			**PBE+*U* (U_{eff}=3.6)**			**PBE+*U* (U_{eff}=4.0)**		
	E_{FM}	E_{AFM}	E_{FM}-E_{AFM}	E_{FM}	E_{AFM}	E_{FM}-E_{AFM}	E_{FM}	E_{AFM}	E_{FM}-E_{AFM}
**No-vdW**	-33.1357	-33.2182	0.082	-30.2398	-30.2426	0.003	-29.9607	-29.9606	-0.0001
**DFT-D2**	-33.9423	-34.0210	0.079	-31.0481	-31.0447	-0.003	-30.7692	-30.7636	-0.0056
**DFT-D3**	-33.8707	-33.9509	0.080	-30.9797	-30.9757	-0.004	-30.7006	-30.6944	-0.0062
